# Electrocardiography Abnormalities in Macaques after Infection with Encephalitic Alphaviruses

**DOI:** 10.3390/pathogens8040240

**Published:** 2019-11-16

**Authors:** Henry Ma, Jeneveve D. Lundy, Katherine J. O’Malley, William B. Klimstra, Amy L. Hartman, Douglas S. Reed

**Affiliations:** Center for Vaccine Research, University of Pittsburgh, 3501 Fifth Avenue, Ste. 9052, Pittsburgh, PA 15213, USA; hem53@pitt.edu (H.M.); jdl68@pitt.edu (J.D.L.); kjo21@pitt.edu (K.J.O.); klimstra@pitt.edu (W.B.K.); hartman2@pitt.edu (A.L.H.)

**Keywords:** alphavirus, vaccine, arbovirus, animal models, nonhuman primates, electrocardiography, ECG, aerosol, encephalitis, equine

## Abstract

Eastern (EEEV) and Venezuelan (VEEV) equine encephalitis viruses (EEVs) are related, (+) ssRNA arboviruses that can cause severe, sometimes fatal, encephalitis in humans. EEVs are highly infectious when aerosolized, raising concerns for potential use as biological weapons. No licensed medical countermeasures exist; given the severity/rarity of natural EEV infections, efficacy studies require animal models. Cynomolgus macaques exposed to EEV aerosols develop fever, encephalitis, and other clinical signs similar to humans. Fever is nonspecific for encephalitis in macaques. Electrocardiography (ECG) metrics may predict onset, severity, or outcome of EEV-attributable disease. Macaques were implanted with thermometry/ECG radiotransmitters and exposed to aerosolized EEV. Data was collected continuously, and repeated-measures ANOVA and frequency-spectrum analyses identified differences between courses of illness and between pre-exposure and post-exposure states. EEEV-infected macaques manifested widened QRS-intervals in severely ill subjects post-exposure. Moreover, QT-intervals and RR-intervals decreased during the febrile period. VEEV-infected macaques suffered decreased QT-intervals and RR-intervals with fever onset. Frequency-spectrum analyses revealed differences in the fundamental frequencies of multiple metrics in the post-exposure and febrile periods compared to baseline and confirmed circadian dysfunction. Heart rate variability (HRV) analyses revealed diminished variability post-exposure. These analyses support using ECG data alongside fever and clinical laboratory findings for evaluating medical countermeasure efficacy.

## 1. Introduction

Equine encephalitis viruses (EEVs) constitute a subset of the alphavirus genus of Family *Togaviridae*. The alphaviruses are grouped into New World and Old World viruses, which generally correspond with groups of signs of clinical disease; New World alphaviruses are more likely to cause encephalitis while Old World viruses tend to cause arthralgia [1,2,3,4]. In the 1930s, these mosquito-borne viruses were among the first alphaviruses to be discovered, and both the United States and Soviet Union experimented with Venezuelan Equine Encephalitis (VEEV) as a potential biological warfare agent after it was realized that VEEV was highly infectious when aerosolized [5,6,7,8,9,10]. These pathogens remain important medical and veterinary pathogens to this day [11].

In the clinical context, encephalitis comprises infection or inflammation of brain parenchyma that may produce signs of diagnosable disease [12]. EEVs can produce a febrile course, encephalitis, and neurological presentations that include seizures, tremor, and photophobia [1,12]. Other symptoms seen in EEV-induced disease includes nausea, vomiting, diarrhea, focal neurological deficits, or altered mental status [13,14,15,16,17,18]. VEEV and western equine encephalitis virus (WEEV) are only rarely fatal in naturally-transmitted human cases, and then typically only in very young or elderly patients [15,19,20,21,22,23,24]. North American isolates of EEEV have a high case fatality rate, estimated to range from 30–70%, although sub-clinical disease may be underreported [13,25]. Survivors of virulent infections can develop persistent neurologic sequelae that affect motor function. Pathological evaluation can confirm or rule out encephalitis by examination of tissue samples for cellular inclusion bodies and lymphocytic infiltrates, and immunohistochemical staining; however, the post-mortem nature of these procedures limits their clinical applicability [26]. Apart from a thorough patient history, confirmation of EEVs as the cause of disease requires assaying for anti-EEV IgM or IgG antibodies in plasma or cerebrospinal fluid (CSF) [13,18,27].

No consensus governs the case management of EEV infections and no licensed treatments or vaccines are available to mitigate an outbreak or intentional release [1,28]. Because aerosol dissemination is not a natural route of transmission, determining efficacy of countermeasures against aerosolized EEVs in a clinical trial is neither logistically nor ethically possible. Licensure of such countermeasures can proceed only in accordance with the FDA Animal Rule, which facilitates pivotal efficacy studies to be conducted in well-characterized animal models that meet appropriate criteria [29]. These criteria require that the disease and pathophysiology in the animal model must resemble what is known to occur in humans. To evaluate the efficacy of a therapeutic treatment against viral encephalitis, biomarkers that correspond with viral penetration into the CNS and/or neurological disease severity are needed. For EEVs, infection of the cynomolgus macaque closely resembles the disease seen in humans [30,31]. Fever was the predominant physiological sign observed in past EEV studies, but it is not clear if this corresponds with a response to peripheral viral replication or viral penetration into the CNS [18,32,33,34]. In VEEV infection of humans and macaques, the febrile response is biphasic; the consensus in the field has been that the first fever likely represents peripheral viral replication while the second fever coincides with viral penetration into the CNS. In macaques, the second febrile period is associated with neurological signs indicative of encephalitis [35]. In contrast, EEEV infections do not have a fever in the initial period seen with VEEV but fever onset for EEEV roughly corresponds with the second fever peak for VEEV and onset of neurological signs in macaques [36].

Previous work with macaques found that heart rate increased in association with fever onset after infection with EEEV [35,36]. A number of viral infections can cause changes in cardiac physiology, some of which appear on electrocardiography (ECG). For instance, myocarditis instigated by viruses—such as Coxsackie B virus, adenovirus, or influenza A—can cause downstream effects that result in the pathogenesis of dilated cardiomyopathy [37]. Multiple examples of diagnostic features of dilated cardiomyopathy through electrocardiography abound in the literature [38,39]. Moreover, other known examples of cardiac pathology visualized by ECG include QT-interval prolongation as a sign of autonomic neuropathy in HIV-infected individuals, though the question of whether the finding represents a consequence of prolonged infection or prolonged courses of treatment with highly active antiretroviral therapy remains to be seen [40,41]. The rationale for investigating cardiac pathology of neurogenic origin stems from the wide variety of infectious agents including diseases of bacterial, viral, and parasitic origin in which pathogenesis, or the host response to disease pathogenesis, can induce a neurogenic basis for cardiac changes [42,43]. In the context of neuroinvasive, encephalitic EEVs, EEEV, and WEEV infect both neurons and cardiac myocytes in avian species and murine models respectively, while focal cardiac lesions have been previously documented in macaques infected with VEEV [44,45,46].

Exploration of a wider range of ECG metrics leverages the advantages inherent in continuous radiofrequency telemetry monitoring equipment. In this study, we hypothesized that physiological changes encountered in macaques following infection with EEEV or VEEV would produce measurable changes in electrocardiographic metrics that would precede or accompany the onset of febrile or encephalitic disease. The focus of this paper rests less on finding diagnostic biomarkers specific for EEEV or VEEV, but rather on the evaluation of the utility of ECG changes as a biomarker of CNS disease and outcome in alphavirus encephalitis in the cynomolgus macaque. We performed repeated measures ANOVA and multiple comparisons of electrocardiographic metrics between pre-exposure, post-exposure, and febrile periods for macaques that developed fever, and recovery periods for macaques that survived febrile courses. Heart rate variability (HRV) was analyzed between days from the baseline period through the post-infection period. Finally, frequency-spectrum analysis was performed on the electrocardiography metrics. Findings indicated that changes in QRS complexes, QT-interval, and RR-interval, among other metrics, could serve as sentinels of EEV-induced disease. 

## 2. Results

### 2.1. ECG Changes in EEEV-Infection of Macaques

**Figure 1:**
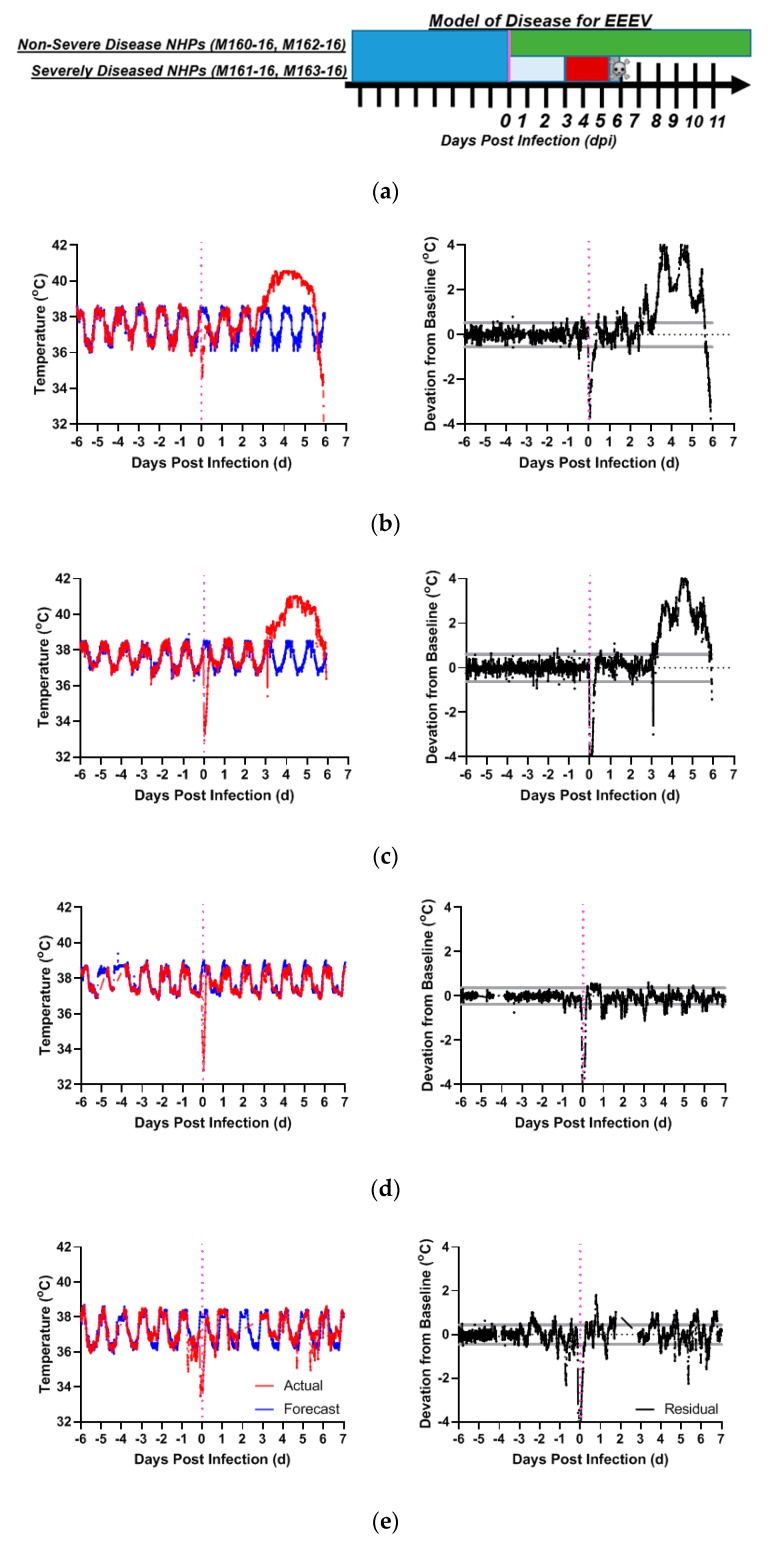
EEEV disease in macaques. Shown are models of disease for (**a**) EEEV-exposed macaques with non-severe and severe courses of alphavirus disease over days post-infection (x-axis). Deep blue denotes pre-infection baseline period, vertical magenta line denotes time of infection. Green bar denotes post-infection period in animals without severe/fatal disease. For animals with severe/fatal disease, powder blue defines post-infection period before the onset of fever, and red signifies the febrile post-infection period. Skull and crossbones mark the mean time to death. Actual and forecasted temperature profiles for two macaques with severe disease requiring euthanasia: (**b**) M161-16, and (**c**) M163-16, compared to macaques without severe disease: (**d**) M160-16 and (**e**) M162-16. Red: actual temperature (°C). Blue: forecasted temperature (°C) from ARIMA modeling. Black: Residual Values (°C) (deviations from forecasted baseline). Gray Lines: upper/lower bounds for residuals. Magenta: aerosol challenge.

To explore the utility of monitoring ECG changes associated with EEEV-induced viral encephalitis caused by New World-alphaviruses, four macaques were exposed to small-particle (≤5 µm mass mean aerodynamic diameter) aerosols containing EEEV. The outcome and the febrile response after infection of the macaques with EEEV are shown in Table 1. Macaques exposed to EEEV manifested either severe or non-severe courses of disease (Figure 1a). ‘Severe disease’ was defined as a course that precipitated moribund status requiring euthanasia. Aerosol exposure to EEEV produced severe disease courses in the two macaques (M161-16, M163-16) that received more than 2.22*10^7^ PFU. In contrast, the two macaques (M160-16, M162-16) that received doses below this amount survived without fever. The appearance of abnormal ECG and fevers manifested only in the macaques with severe disease that eventually met euthanasia criteria.

In the macaques with severe EEEV disease (M161-16, M163-16), the disease course replicated with high fidelity what was reported previously with a different EEEV strain [36]. The macaques became febrile post-challenge at three days, culminating in a temperature plateau of 40–41°C around 4.0 days post-infection (dpi) (Figure 1b,c). This plateau was followed by a terminal decline in temperature beginning at approximately 5 dpi, heralding a moribund state occurring at approximately 6 dpi. For macaques with severe disease, the time between 0–3 dpi defined the post-infection period and the time following 3 dpi denoted the febrile period. None of the EEEV-infected macaques with severe disease survived, so a recovery period was not defined. For the two macaques that survived lower doses of EEEV there was no febrile response post-infection (Figure 1d,e) nor any changes in behavior or activity or any other clinically apparent sequelae.

**Figure 2:**
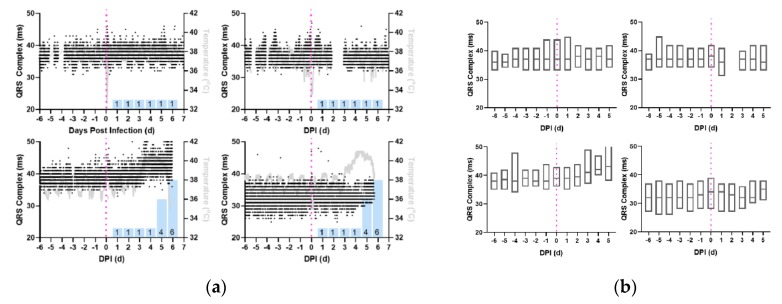
Increase in QRS complex during the febrile period in macaques with severe EEEV. Shown are the profiles (**a**) of QRS complex derived from 1-min average measurements for four individual macaques infected with EEEV. Black: QRS Complex (ms), Gray: Temperature (°C), and Magenta: aerosol challenge. Blue Bars: Neurological score on corresponding day. (**b**) Average daily QRS complex measurements, from repeated measures ANOVA, depicted in with boxes delimited by interquartile range and center line signifying median value. For (**a**) and (**b**), Top Left: M160-16, Bottom Left: M161-16, Top Right: M162-16, Bottom Right: M163-16.

In the immediate post-infection period, the profile of QRS Complex data show no readily apparent changes between 0 dpi to 3 dpi, in either the directly observable trace (Figure 2a) or in daily aggregates of the QRS complex (Figure 2b). Beginning with fever onset on 3 dpi, the QRS complex exhibited progressive increases in severely diseased EEEV-infected macaques which continued until euthanasia criteria were met (Table 2, *p* < 0.05). Macaques exposed to the lower doses of EEEV displayed no significant deviations from their baseline QRS complexes (Figure 2b).

**Figure 3:**
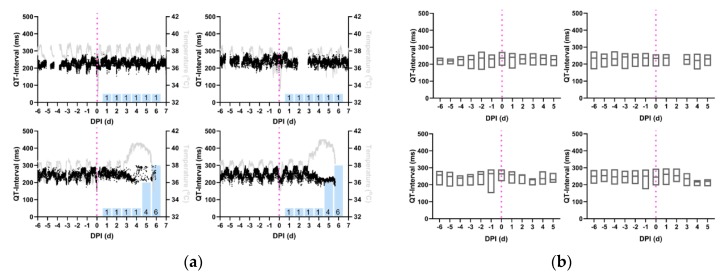
Reduction in QT-interval during the febrile period in macaques infected with severe EEEV. Shown are the profiles (**a**) of QT-interval derived from 1-min average measurements for four individual macaques infected with EEEV. Black: QT-interval (ms), Gray: temperature (°C), and Magenta: aerosol challenge. Blue Bars: Neurological score on corresponding day. (**b**) Average daily QT-interval measurements, from repeated measures ANOVA, depicted in with boxes delimited by interquartile range and center line signifying median value. For (**a**) and (**b**), Top Left: M160-16, Bottom Left: M161-16, Top Right: M162-16, Bottom Right: M163-16.

Similar to the QRS-interval, there are no visually apparent changes for any of the EEEV-infected macaques in the QT-interval trace (Figure 3a) or the daily QT-interval aggregate (Figure 3b) in the post-infection period until 3 dpi. With the onset of fever on 3 dpi, the two macaques with severe EEEV disease exhibited shortened QT-intervals and a loss of diurnal variation. This continued until the macaques were moribund (Table 3, *p* < 0.05). A false positive result surfaced from the repeated measures ANOVA for M160-16, one of the macaques without severe disease, but this result is attributable to data loss during baseline collection; (Figure 3a) this effect disappears once the days containing missing baseline data are censored. The missing diurnal baseline data for 160-16 rendered an apparent increase in QT-interval on the initial analysis. In the macaques exposed to lower doses of EEEV that did not develop clinical disease, no reductions or loss of diurnal variation in QT-interval were seen in the study period (Figure 3b).

**Figure 4:**
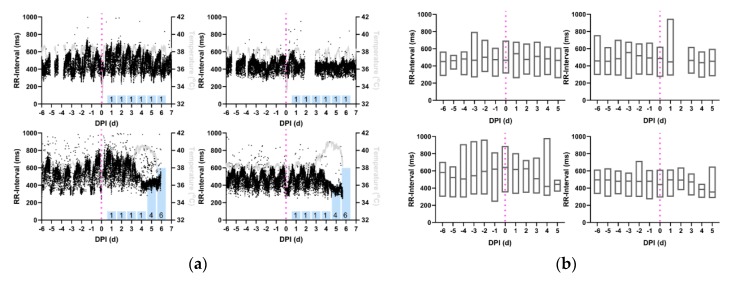
Reduction in RR-interval during the febrile period in macaques infected with EEEV. Shown are the profiles (**a**) of RR-interval derived from 1-min average measurements for four individual macaques infected with EEEV. Black: RR-interval (ms), Gray: temperature (°C), and Magenta: aerosol challenge. Blue Bars: neurological score on corresponding day. (**b**) Average daily RR-interval measurements, from repeated measures ANOVA, depicted in with boxes delimited by interquartile range and center line signifying median value. For (**a**) and (**b**), Top Left: M160-16, Bottom Left: M161-16, Top Right: M162-16, Bottom Right: M163-16.

RR-intervals in macaques infected with EEEV follow a similar pattern to that seen with the QT-interval (Figure 4a). From challenge to 3 dpi, no visually apparent changes occurred in the RR-interval in any of the macaques. On 3 dpi, the severely-infected macaques had decreases in both the trace and the median RR-interval in aggregate data (Figure 4b). No changes in RR-interval were observed for macaques with nonsevere courses of disease (Table 4, *p* < 0.05).

**Figure 5:**
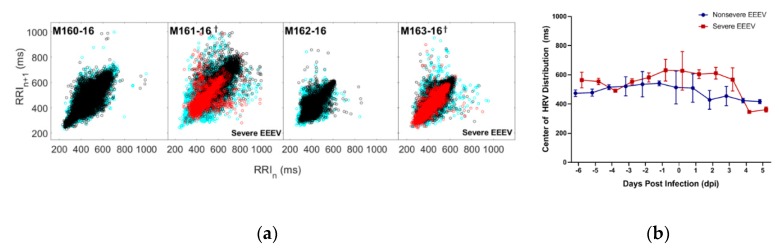
Heart rate variability decreases during the febrile period in macaques infected with EEEV. (**a**) Distributions of heart rate variability of each EEEV-infected macaque are plotted in aggregate and color-coded by disease period (Cyan: Pre-Infection, Black: Post-Infection, Red: Febrile (if applicable)). (**b**) Repeated measures ANOVA of HRV data for macaques with nonsevere and severe EEEV infection (*p* < 0.05). Central tendency of macaques with severe disease (Red) significantly diminishes after 3 dpi, compared to macaques with nonsevere disease (Blue).

Heart rate variability was plotted as ordered pairs of the RR-interval data collected at time *n* (RRI_n_) and RR-interval data collected at time *n+1* (RRI_n+1_), wherein *n* represents an integer value in corresponding to a minute on the macaque’s time scale. In aggregate, when these scatter plots are superimposed and color-coded according to pre-infection, post-infection, and febrile disease periods (Figure 5a), there is a visually notable contraction in the distribution of ordered pairs during the febrile period in the severely diseased macaques. When decomposed into Poincaré plots to showcase the day to day distribution of HRV, with the central tendency of the plots represented by the geometric mean (Supplemental Figures S1 and S2), the disease courses of the severely diseased macaques demonstrate (Figure 5b) a statistically significant decrease in the geometric mean of the distribution (*p* < 0.05) with an accompanying diminishment of variability, which recapitulates the markedly reduced heart rate variability roughly corresponding to the results seen in Figure 4.

### 2.2. ECG Changes in VEEV-Infection of Macaques

**Figure 6:**
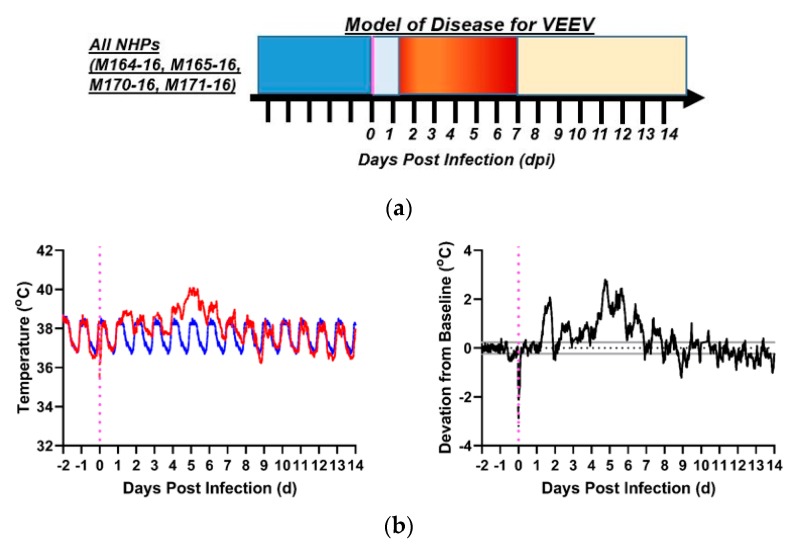

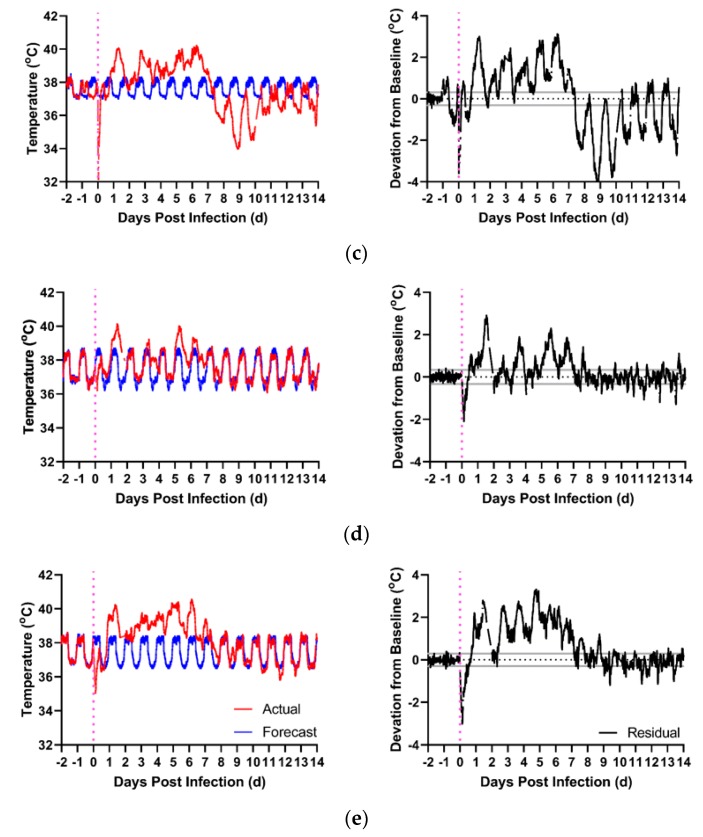
VEEV disease in macaques. Shown is the model of disease for (**a**) VEEV-exposed macaques over days post-infection (x-axis); all macaques developed febrile encephalitic disease. Deep blue denotes pre-infection baseline period, vertical magenta line denotes time of infection, powder blue defines post-infection period before the onset of fever, red signifies the febrile post-infection period, and yellow signifies recovery period after febrile illness subsides. Actual and forecasted temperature profiles for female macaques: (**b**) M164-16, (**c**) M165-16, and male macaques: (**d**) M170-16 and (**e**) M171-16. Red: actual temperature (°C); Blue: forecasted temperature (°C) from ARIMA modeling, Black: residual values (°C) (deviations from forecasted baseline). Gray Lines: upper/lower bounds for residuals. Magenta: aerosol challenge.

Aerosol parameters of VEEV-infected macaques are summarized in Table 1. Exposure to VEEV produced a characteristic biphasic febrile illness in all exposed macaques with the first phase beginning within 18–24 h post-infection and the second phase within 1–3 days after the first phase had resolved (Figure 6a). Though fever profiles of the several macaques in this cohort did not match precisely, they were similar enough as to fit the model. All of the macaques challenged with VEEV recovered from these fevers, and none of the macaques became so ill as to meet euthanasia criteria. Post-exposure, macaques became febrile after a latency period of a day, with a febrile peak of 40–41 °C at approximately 1.3 dpi (Figure 6b–e). The temperature returned to normal by 1.5 dpi. A second fever occurred at 2.5 dpi and lasted approximately four days until 6.5–7.0 dpi. Subsequently, the macaques’ temperatures returned to normal, with the exception of hypothermia that occurred in M165-16. For analysis of VEEV-infected macaques, the post-infection period was defined as 0–1 dpi (inclusive), the febrile period as 2–7 dpi (inclusive), and the recovery period as 8 + dpi.

**Figure 7:**
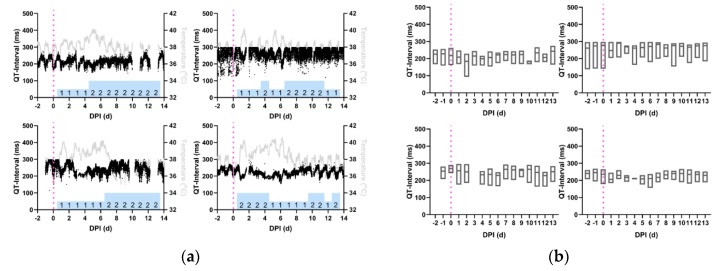
Reduction in QT-interval during the febrile period in macaques infected with VEEV. Shown are the profiles (**a**) of QT-interval derived from 1-min average measurements for four individual macaques infected with VEEV. Black: QT-interval (ms), Gray: temperature (°C), and Magenta: aerosol challenge. Blue bars: neurological score on corresponding day. (**b**) Average daily QT-interval measurements, from repeated measures ANOVA, depicted in with boxes delimited by interquartile range and center line signifying median value. For (**a**) and (**b**), Top Left: M164-16, Bottom Left: M165-16, Top Right: M170-16, Bottom Right: M171-16.

QT-intervals of the VEEV-infected macaques decreased in the immediate post-infection period and this decrease persisted through the febrile period. Examination of QT-interval traces (Figure 7a) reveals that in two macaques (M165-16, M171-16) the decreased QT-interval persisted through the recovery period, but for the other two in the cohort (M164-16, M170-16), the QT-interval exhibited a return to baseline values during recovery. The daily QT-interval aggregate (Figure 7b) demonstrates that the QT-interval exhibited a sustained decrease beginning from the immediate post-infection period through the febrile period, until the beginning of the designated recovery period at 8 dpi; repeated measures ANOVA indicated statistically significant departures from baseline QT-interval in all four macaques (Table 3, *p* < 0.05). 

**Figure 8:**
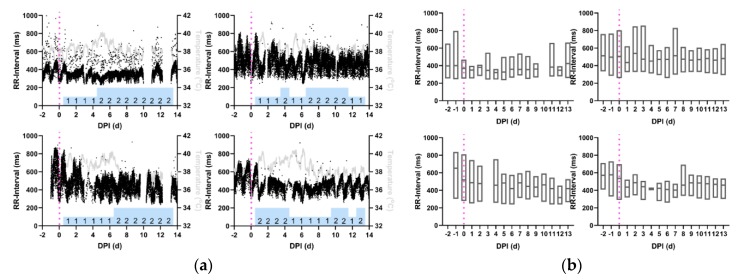
RR-interval decreases and remains decreased in macaques with VEEV encephalitis. Shown are the profiles (**a**) of RR-interval derived from 1-min average measurements for four individual macaques infected with VEEV. Black: RR-interval (ms), Gray: temperature (°C), and Magenta: aerosol challenge. Blue Bars: neurological score on corresponding day. (**b**) Average daily RR-interval measurements, from repeated measures ANOVA, depicted in with boxes delimited by interquartile range and center line signifying median value. For (**a**) and (**b**), Top Left: M164-16, Bottom Left: M165-16, Top Right: M170-16, Bottom Right: M171-16.

The RR-intervals of macaques infected with VEEV (Figure 8a) demonstrated decreases in all macaques beginning immediately post-infection and persisted in the same manner throughout recovery. This effect is visible in the median RR-interval in aggregate data (Figure 8b), in which the RR-interval can be seen to trend downward beginning in the immediate post-infection period at 0 or 1 dpi (Table 4, *p* < 0.05).

**Figure 9:**
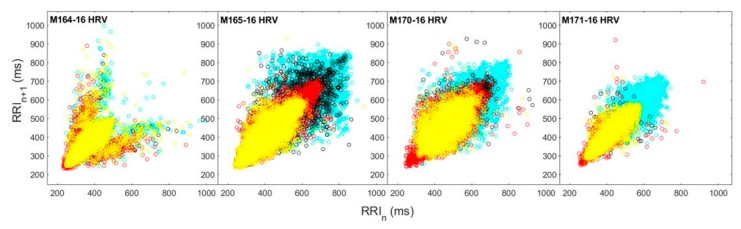
Heart rate variability decreases during the febrile period in macaques infected with VEEV. Distributions of heart rate variability of each VEEV-infected macaque are plotted in aggregate and color-coded by disease period (Cyan: pre-infection, Black: post-infection, Red: febrile, Yellow: recovery).

The HRV for the VEEV-infected macaques show in aggregate that when scatter plots of daily HRV distributions are superimposed and color-coded according to pre-infection, post-infection, febrile, and recovery periods (Figure 9), the distribution of ordered pairs contracts, and remains contracted well after the febrile period. When decomposed into Poincaré plots by day post infection, representative of each disease period (Supplemental Figure S3), the persistence of the decrease in heart rate variability is even more apparent. As with the related RR-interval, all macaques experienced this effect.

### 2.3. Frequency Spectrum Analysis

Using fast Fourier transformation (FFT), the differences between the outlined periods became apparent in the macaque subjects with severe disease. The first local maximum in the frequency-transformed data, known as the fundamental frequency, of each metric was computed in cycles per day (cpd). Patterns of change in the frequency spectra varied to a minimal degree between different electrocardiographic metrics, with particular regard to differences between those metrics that co-varied; for example, metrics that exhibited a high degree of covariance with heart rate, such as RR-interval, exhibited similar trends with respect to how the fundamental frequencies changed between pre-, post-, and febrile periods. The amplitude of the y-axis data in the frequency plots, rendered in units of magnitude (ms^2^) depended largely on the number of points in and the amplitude of the time-domain signal. Fundamental frequencies for the RR-interval, as a representation of the overall trend of frequency changes, are tabulated in Table 5.

Prior to infection, the fundamental frequency for RR-interval was different between macaques; however, most fell into a fairly narrow range. Macaques infected with EEEV that did not develop severe disease saw no significant changes in RR-I fundamental frequencies post-infection (Table 5). Changes in RR-I fundamental frequencies were much larger in severely diseased macaques. In the EEEV-infected macaques with severe disease, the post-infection frequency increased to 0.879 and 0.769 cpd respectively then declined to 0.571 and 0.549 cpd in the febrile period although this was still elevated compared to baseline. Values for VEEV were larger due to the extended time periods used for analysis. Examination of fundamental frequencies identified high fundamental frequency values in the VEEV-infected macaques with differences based on sex: 5.97 cpd and 11.25 cpd for females and males respectively, at pre-infection. A statistically significant effect was found for the influence of sex on HRV; though some differences may appear visually, follow-up investigation (Supplemental Figure S4) by a two-factor ANOVA demonstrated that these differences were not spurious in nature. However, for both females and males, the fundamental frequencies increased after VEEV infection and then dropped below baseline levels in the febrile period with the sharpest decline in the male macaques. With the onset of the designated recovery period in VEEV, the fundamental frequency of the latter period, when body temperatures largely returned to normal, the fundamental frequencies in the VEEV-infected macaques remained lower than pre-infection although the greatest reduction was seen in the males.

## 3. Discussion

### 3.1. QRS Complexes

The QRS complex represents the ventricular depolarization of the heart and therefore the speed with which ventricular contraction occurs; therefore, an increased duration of the QRS complex could indicate imbalances of blood chemistry, with hypokalemia as an example. The clinical observations taken suggested the feasibility of such a mechanism, since both severely diseased EEEV-infected macaques had decreased water intake during the febrile period. However, when analysis was performed of blood chemistry samples taken pre-infection and post-infection this hypothesis was discarded due to the normal potassium values (3.5–5 mEq), leaving the question open as to whether autonomic dysregulation was responsible for the pathological phenomenon observed. The lack of blood chemistry between infection and necropsy means that the possibility of electrolyte balance dysregulation cannot be completely ruled out. Recent investigation has suggested that autonomic modulation of heart rate can manifest changes in the shape of the QRS complex, with shorter QRS complexes suggestive of an increase in sympathetic activity [47]. However, in the macaques in which this occurred, the finding was more likely a covariate of an increased heart rate due to febrile illness.

### 3.2. QT-Intervals

The QT-interval represents the time interval between the Q-wave (beginning of ventricular depolarization) and the T-wave (ventricular repolarization) of the ECG. The finding of decreased QT-intervals in the EEEV-exposed macaques with severe disease, again, represents an anomaly in that though the QT-interval is decreased, the QRS complex, which comprises a segment of the QT-interval, is increased. The QT-interval is of particular interest for the testing of therapeutic compounds, due to the extent to which certain small molecule drug candidates can affect the QT-interval. Ion channels in the myocardium can be affected by off-target effects, and this represents concern for potential pharmacological intervention [48]. However, pre-clinical studies in uninfected animal models should mitigate the potential for such an eventuality. Perhaps the most thought-provoking aspect of the QT-interval effects resides in the alterations to diurnal variation during and sometimes after the period of febrile illness, a finding mirrored in the upcoming discussion of the RR-interval and heart rate variability. 

### 3.3. RR-Interval, Heart Rate Variability, and Frequency Spectrum Analysis

The RR-interval denotes the actual interval of time between two heartbeats, while HRV signifies the variation the former measure, expressed in milliseconds [49]. The notion that RR-interval and HRV can illustrate the circadian rhythm of autonomic regulation has been well characterized [50,51,52]. Changes in HRV have comprised the subject of study by multiple authors who have associated alterations in the RR-interval and HRV with pathology such as diabetes mellitus and its associated diabetic neuropathy, heart failure, myocardial infarction, and other chronic conditions [50,53,54]. Additionally, more recent studies have suggested a link not only between inflammatory markers and HRV [55], but also between changes in the RR-interval and heart rate variability in a number of diseases of infectious origin [56,57,58].

For both VEEV-infected and severely EEEV-infected macaques, there was a decrease in the RR-interval in the febrile period. For severe EEEV-infection, the decrease was greater, with a more complete loss in diurnal variation. Subsequent work with macaques challenged with EEEV (not published) demonstrates that the EEEV macaques with no overt clinical signs of disease are indeed infected, as determined by plaque assay and antibody neutralization titers from plasma obtained from serial blood draws following the aerosol challenge. Therefore, as the EEEV-challenged macaques with nonsevere disease also have limited to no changes in RR-interval as depicted in daily Poincaré plots, this effect can therefore distinguish severe from nonsevere courses of disease. RR-interval remained persistently low in all four VEEV-infected macaques even through the recovery period. Despite recovery from encephalitic disease, the results suggest that infection with VEEV may have altered the homeostatic set point of autonomic regulation, as previously reported in the literature [12,18,59]. 

The fundamental frequency encapsulates the periodicity of the original time domain signal (the ECG metric), typically multiple times per day due to idiosyncrasies in the autonomic regulation of cardiac function. Although the fundamental frequency is what was used as the benchmark, subsequent local maxima are registered as *nth* harmonics, or subsequent echoes, of the fundamental frequency. The frequency data over the sequential periods of EEV disease courses suggests similar trends in disease courses; post-infection, the fundamental frequencies of the ECG metrics typically increase, then decrease during the febrile period in individuals with severe disease. 

In the context of frequency spectrum analysis, for macaques with severe courses of EEEV, the fundamental frequencies of the RR-interval increased between pre-infection, the latency period, and febrile disease, whereas the fundamental frequencies of macaques with nonsevere courses of EEEV remain relatively unchanged. This result corroborates the value of RR-interval in distinguishing between severe and nonsevere cases of EEEV and highlights the capacity of frequency spectrum analysis to perform this distinguishing function. For VEEV, the fundamental frequencies of the RR-interval increased post-infection during the short latency period; with the onset of fever, the fundamental frequency decreased. The fundamental frequency of the RR-interval again decreased during recovery, and the permanence of the decreased RR-interval, plotted in Poincaré plots in Supplemental Figure S3, and diminished HRV for the VEEV-exposed cohort is recapitulated in Table 5 by the frequency spectrum analysis, suggesting a real physiological rather than artifactual basis for these results. The existence of post-infection changes in the fundamental frequencies in subjects who survived EEEV challenge suggests that neurological sequelae could occur even without a strong febrile response or obvious clinical signs, sequelae that could have long term health consequences. 

### 3.4. Limitations and Future Work

We do acknowledge that there is a shortfall in investigating the dose–response relationship of encephalitic alphavirus disease through electrocardiographic means because of the small sample sizes of the macaque cohorts, a limitation partially offset by the sheer volume of data obtained from each subject. The potential confounding effect of subject sex likewise presents a limitation; the EEEV cohort was entirely male, while the VEEV cohort was split between the sexes. Baseline metrics varied significantly between individual macaques, although all baseline measures fell within normal limits. At this juncture, it is not clear whether the results reported here are applicable to other viral encephalitides. Direct application of these analyses in a human clinical context may suffer from limited generalizability, based on the variability in baseline ECG metrics between individual macaques and baseline ECG data would not be available for most human patients.

There are a number of remaining questions, including the following: do differences in ECG metrics reflect sex-based differences in host responses to inhalational alphavirus infection? If so, would similar effects be seen in the vein of periodicity related to the primate estrus cycle, especially with respect to the loss of circadian variation seen in all signals? More information is required to perform an analysis of whether the lung morphology or body weight might alter the effects seen in the ECG metrics in not only pre-infection data, but in all disease periods. A decrease in RR-interval during the febrile period appears to be a generalizable phenomenon to other severe, acute infectious diseases (D.S. Reed, manuscript in preparation) although it is not clear if these other severe infections penetrate the CNS particularly when the route of exposure is also aerosol. The other parameters noted here (QT-interval, QRS) may be specific to viral encephalitis. Finally, whether a dose–response relationship truly governs the appearance of changes in ECG metrics, or whether those effects observed are binary once exposure exceeds a threshold dose remains to be further investigated. The results described appear to support the latter theory; while these results may not be specific to viral encephalitides and may not supplant fever as a biomarker for the initiation of treatment, they may serve as useful predictors of outcome. Further investigation with electroencephalography can better determine whether the differences observed between sexes are definitively responsible for the dichotomies observed in the results, and can help to establish whether the observed ECG effects stem from a basis in autonomic dysregulation, from direct viral infection, the immune response to infection, or a combination of these factors.

Future studies with the telemetered macaque model may improve upon the utility of ECG data with respect to the prognostication of disease outcome, with additional statistical methods such as principal component analysis holding much promise for such an application. In conjunction with analysis of electroencephalography and other telemetric modalities, such methods could prove a powerful tool, followed in real time or quasi-real-time by lab staff, for investigating both viral pathogenesis and developing therapeutics and countermeasures against EEV aerosol infection. Whether EEVs directly affect cardiac responses as captured by electrocardiography metrics with respect to damage to the myocardial syncytium versus the disruption of autonomic outflow requires further study and remains an area of continuing investigation. The main focus of interest in this macaque model of alphavirus encephalitis remains centered about the disease phenotypes observed for inhalational EEEV and VEEV; the manifestation or abrogation of these phenotypes can ultimately contribute to an evidence base to determine whether a medical countermeasure candidate has achieved clinical efficacy.

## 4. Materials and Methods

### 4.1. Statement on Rationale, Use, and Care of Animals

This work received approval from the University of Pittsburgh Institutional Animal Care and Use Committee (IACUC) and adhered fully to all stipulations of the Animal Welfare Act Regulations and the Guide for the Care and Use of Laboratory Animals [60]. Specifically, this work was performed at the Regional Biocontainment Laboratory (RBL) located at the University of Pittsburgh which is itself accredited by the Association for Assessment and Accreditation of Laboratory Animal Care (AAALAC). The rationale for the development of a nonhuman primate (NHP) model of inhalational EEV disease fulfills the necessity criteria outlined by the FDA Animal Rule [29,61,62]. The cynomolgus macaque (*Macaca fascicularis*) has served as a model organism for EEVs since the discovery of these viruses in the 1930s [6,63]. Additionally, this species provides distinct advantages over rodents due to the greater anatomical and physiological similarity of macaques to humans [35,36,64,65]. Infection of macaques through exposure to infectious aerosols of EEVs reproduces CNS lesions similar to those produced by EEV infection in human cases [35,36,66,67]. The augmentation of this well-established animal model for respiratory infection to produce encephalitis recapitulates the hallmarks of encephalitic disease with respect to histopathology and traditionally-used physiological biomarkers towards the development of improved vaccines against aerosol-induced disease.

Cynomolgus macaques were singly housed for the duration of these studies. Subjects were monitored on a daily basis and clinically scored according to a series of ordinal scales for the following categories: neurology, activity, and temperature [35,36,68]. The neurological scoring scale ranged from 1–6 and accounted for signs such as tremor, gait imbalance, nystagmus, head pressing, seizures, and coma. The activity score ranged from 1–6 and accounted for mental status as determined by posture, facial expressions, responses to stimuli, and interactions with observers. Finally, the temperature scale ranged from 1–6, accounted for core temperature, and gauged for fever and hypothermia. The ordinal scoring scales were summed and the cumulative score was used to determine whether a macaque warranted more frequent observation or was at risk of imminent death which would require immediate euthanasia. Excreta and food/fluid intake were also monitored daily.

### 4.2. Animal Model Telemetry Implantation 

The macaques used in this study were implanted intra-abdominally with Data Sciences International (DSI, St. Paul, MN) radiofrequency transmitters (DSI model mo. M01) by a DSI veterinarian at Covance Laboratories (Princeton, NJ). In preparation for surgery, the macaques were each administered 25 mg/kg cefazolin (1^st^-generation cephalosporin) for infection control and 1 mg/kg ketoprofen for analgesia, and the incision site, 5 cm superior to the anterior superior iliac spine, was shaved. Macaques were anesthetized with 10 mg/kg ketamine with continuous IV administration of 3% normal saline via the great saphenous vein. Isoflurane (2% v/v) was then administered for continuous anesthesia with 4.0–4.5 L/min of O_2_. The incision site was draped and sterilized with chlorhexidine antiseptic and 70%v/v isopropyl alcohol. A 5 cm incision was made parallel to the midline, into which the implant was inserted. ECG leads sutured with one lead placed under the right pectoralis muscle and the other placed parallel to the left inguinal region to approximate ECG Lead II placement as in the 12-lead ECG format [69]. The incision was closed with a simple interrupted stitch. Post-operatively, each macaque was eligible for 3 days’ administration (PRN) of buprenorphine for analgesia and received 5 days’ administration (BID) of cefazolin for infection control.

### 4.3. Virus Culture and Dose Determination

Virus cultures were conducted as described in the literature [70]. The EEEV and VEEV isolates used in this study were single passages of infectious clones generated from human isolates: V105 (EEEV) and INH-9813 (VEEV-IC). For both alphaviruses, stocks were generated through construction of cDNA libraries; 5′-capped, infectious viral RNA was produced through in vitro synthesis from linearized cDNA plasmid template alphavirus genomes. These genomes were transformed into baby hamster kidney (BHK-21, ATCC) cells by electroporation (Klimstra, manuscript in preparation). Centrifugation of the supernatant clarified this yield 18–24 h post-electroporation, and single-use aliquots were stored at −80 °C and denoted p0 for passage zero. The p0 stocks underwent standard plaque assays with BHK-21 cells for titering and were then used to infect Vero cells (ATCC CCL-81) at MOI 10 in roller bottles. At 1 day post-infection of the Vero cells, the supernatant was decanted and clarified by centrifugation, then purified by means of layering the supernatant over a 20%/60% sucrose cushion prior to ultracentrifugation. The virus, residing in the gap, or interface, between the 20%/60% sucrose cushion was collected then diluted in 10 mM Tris, 1mM EDTA, 100 mM NaCl–STE 10X NaCl (TNE) buffer. The resultant mixture was again laid over a 20% sucrose solution and subjected to ultracentrifugation to pellet the virus. The pelleted virus was resuspended in Opti-MEM^®^ Reduced Serum Medium (ThermoFisher, catalog no. 31985-070) and single-use aliquots were stored at −80 °C. The virus stock (passage 1), nebulizer samples, and aerosol samples were titered using standard plaque assays on BHK-21 cells and the aerosol LD_50_ determined in mice before use in macaque studies. The LD_50_ observed for EEEV in macaques was 2.2 × 10^7^ PFU, with the macaque cohort described in the results conforming to this measure.

### 4.4. Aerosol Exposure of Macaques to EEVs

For each exposure, the aerosol generated for challenge was prepared as described in the literature [71]. Aerosol exposures were performed under the control of the Aero3G aerosol management platform (Biaera Technologies, Hagerstown, MD) as previously described [72]. Macaques were anesthetized with 6 mg/kg Telazol^®^ (Tiletamine HCl / Zolazepam HCl). Each macaque was then weighed, bled, and transported to the modified Class III biosafety cabinet using a mobile transport cart. The macaque’s head was placed inside an acrylic head-only exposure chamber. Jacketed External Telemetry Respiratory Inductive Plethysmography (JET-RIP; DSI) belts were placed around the abdomen and chest of the macaque and calibrated to a pneumotach. This allowed monitoring and recording of respiratory function via the Ponemah software platform (DSI) during the aerosol [73]. EEV aerosols were generated using an Aeroneb vibrating mesh nebulizer (Aerogen, Chicago, IL) as previously described [74]. Exposures were 10 min in duration. To determine inhaled dose, aerosol sampling was performed during each exposure with an all-glass impinger (AGI; Ace Glass, Vineland, NJ). Particle size was measured once during each exposure at 5 min using an aerodynamic particle sizer (TSI, Shoreview, MN). A 5-min air wash followed each aerosol before the macaque was removed from the cabinet, and transported back to its cage and observed until fully recovered from anesthesia.

### 4.5. Electrocardiography Data Collection

Electrocardiography, activity, and temperature data were collected continuously from implanted macaques for at least two days before aerosol exposure and for the duration of the disease course post-exposure. An example of a raw ECG trace is shown in Supplemental Figure S5. A computer connected to a communication link controller (CLC; DSI) collected signals from the implanted macaques by way of transceivers mounted in the room. Video cameras were positioned to record macaque behavior and were programmed to record continuously, synchronously, alongside the telemetry data. Because of the amount of telemetry and video data generated daily, the system had to be manually stopped and restarted every morning. The Ponemah suite (v.5.20SP8; DSI) provides an interface that allows for graphical inspection of data collected in real time from the M01 implants. For analysis, data were read from either the native raw file or reviewed in spreadsheet, statistical analysis, and plotting programs. Temperature and ECG measures were sampled at 10 Hz with a printed value every 5 s [75]. For fever, a Box-Jenkins ARIMA model was used to predict 15-min average body temperature from baseline data; significant deviations (3 times the square root of the residual sum of squares) of actual from predicted temperatures were scored as fever. Fever duration was calculated as the number of fever points divided by 4 (the number of points per hour); fever severity was measured as ‘fever-hours’, the summation of residual elevated temperatures divided by 4. ARIMA analysis was done using Number Cruncher Statistical Systems (NCSS) 2007 software. Electrocardiography metrics studied are displayed in Supplemental Table S2. Standard deviations of these metrics were measured and recorded at the same sampling frequencies as well. 

### 4.6. Electrocardiography Analysis Methods

Disease courses were defined by the appearance of fever during the clinical observation period; for each cohort of EEV-infected macaques, periods were established based on the timing of infection and fever: pre-infection, post-infection (prior to fever onset), febrile period, and recovery, when applicable. These periods were constructed according to the markers for fever explained above. Analyses of electrocardiography metrics were carried out by considering the median daily electrocardiographic metric of interest or by considering the aggregate median electrocardiographic metric of interest over the periods previously defined. 

Electrocardiographic metrics were sampled from the raw data at a rate of one sample per minute for 1440 data points per day to reduce the computational burden on the analytical software and to reduce the impact of noise on producing artificially significant results. Statistical analyses were performed in two stages: preliminary analysis of temperature and activity were performed alongside the collection of data, with limited analysis of the raw ECG traces. A more comprehensive statistical analysis was performed with a within-subjects repeated measures ANOVA approach. Due to significant variability in baseline metrics, the baseline period for each macaque served as its own control for this study. All significance levels consisted of α < 0.05. Heart rate variability was mapped as described by Golinska, and Poincaré plots were constructed according to guidelines described by Henriques et al. [76,77]. 

To examine circadian changes in the ECG data, analysis began with the fast Fourier Transform to render the time-series data into the frequency domain; that is, time-series of the ECG metrics were decomposed into linear combinations of basic trigonometric functions. The frequencies of these trigonometric functions produce a discretized spectrum, composed of each component frequency of the original time-series. The fundamental frequency, or first local maximum in the frequency spectrum window, in units of cycles per day, characterized the changes that occurred between different periods of the disease course. Changes in the fundamental frequency or subcomponents of the normal cyclic rates of ECG metrics were quantified and visualized. These procedures were carried out in MATLAB 2018b (Mathworks, Natick, MA) with the use of onboard algorithms for frequency spectrum analysis. 

## 5. Conclusions

This work was performed to further develop and characterize the macaque model for aerosol exposure to EEVs. In agreement with what we saw previously, fever was a prominent indicator of EEV disease. Across both EEEV and VEEV, it is notable that the macaques who succumbed to severe disease had a maximum temperature difference from predicted of 4.0 °C or higher while all of those that survived infection had maximum temperature differences less than 4.0 °C from predicted. The goal of these studies was to determine whether other physiological parameters might be suitable as biomarkers or indicators for future efficacy studies. The augmented animal model might suffice to evaluate therapeutic compounds under the FDA Animal Rule. Although these are encephalitic viruses, heart function is controlled by the nervous system so we surmised that ECG metrics would be useful for this objective. Indeed, the data suggests that ECG metrics are useful for this purpose, but the ECG metric chosen will be dependent on the particular alphavirus used and will likely need to be paired with body temperature to paint a complete picture of the disease and outcome in macaques. The most prominent set of significant electrocardiographic metrics that distinguish and characterize both EEEV and VEEV disease courses consist of QRS complexes, QT-interval, and RR-interval. 

## Figures and Tables

**Table 1:** EEV courses of infection in macaque cohort.

Virus	Macaque	Sex	Dose *	TTD ^δ^	Neuro ^£^	ΔMax ^γ^	Onset ^γ^	Duration ^γ^	Fever-Hours ^γ^
EEEV	M161-16	M	8.2	6	Y	4.2	3	70.8	144.2
	M163-16	M	7.5	6	Y	4.4	3	65.0	154.9
	Mean		8.0			4.3	3	67.9	149.6
	M160-16	M	7.0	S	N	0.7		14.3	6.5
	M162-16	M	5.7	S	N	1.8		41.5	30.5
	Mean		6.8			1.3		27.9	18.5
VEEV	M164-16	F	7.1	S	Y	2.8	1	187.8	171.6
	M165-16	F	6.9	S	Y	3.1	1	260.5	282.5
	M170-16	M	6.3	S	Y	2.9	1	180.5	153.8
	M171-16	M	6.0	S	Y	3.3	1	238.0	305.9
	Mean		6.8			3.0	1	216.7	228.4

Macaques received aerosols of either EEEV or VEEV. * Doses in log_10_ pfu. ^£^ Neuro = neurological signs (tremors, seizures, photophobia, head pressing), ^δ^ TTD = Time to death, S = Survived to 28 days post infection. ^γ^ Fever data. ^Δ^ Max = maximum difference from predicted; Onset = onset of fever duration > 8 h; Duration = fever duration, in hours; Fever-hours = summation of all significant differences in temperature from predicted. Additional clinical data (e.g., macaque age, weights, plaque reduction neutralizing titer) are in Supplemental Table S1.

**Table 2:** Repeated measures ANOVA statistics for QRS complexes.

Virus	Macaque	Sex	Dose ^£^	TTD ^δ^	F-Statistic	*p*-Value
EEEV	M161-16	M	8.2	6	4.780	0.0346 *
	M163-16	M	7.5	6	4.034	0.0490 *
	M160-16	M	7.0	S	3.833	0.058
	M162-16	M	5.7	S	3.046	0.331
VEEV	M164-16	F	7.1	S	29.55	<0.00001 *
	M165-16	F	6.9	S	164.7	<0.00001 *
	M170-16	M	6.3	S	48.40	<0.00001 *
	M171-16	M	6.0	S	84.17	<0.00001 *

Statistics and *p*-values for QRS complex for EEEV and VEEV-infected macaque cohorts. ^£^ Doses in log_10_ pfu. ^δ^ TTD = Time to death. “S” denotes survivor. * *p*-value lower than significance level α = 0.05.

**Table 3:** Repeated measures ANOVA statistics for QT-interval.

Virus	Macaque	Sex	Dose *	TTD ^δ^	F-Statistic	*p*-Value
EEEV	M161-16	M	8.2	6	6.472	0.016 *
	M163-16	M	7.5	6	24.95	0.00005 *
	M160-16	M	7.0	S	3.884	0.055
	M162-16	M	5.7	S	2.962	0.335
VEEV	M164-16	F	7.1	S	36.30	<0.00001 *
	M165-16	F	6.9	S	62.56	<0.00001 *
	M170-16	M	6.3	S	25.46	<0.00001 *
	M171-16	M	6.0	S	82.35	<0.00001 *

Statistics and *p*-values for QT-interval for EEEV and VEEV-infected macaque cohorts. ^£^ Doses in log_10_ pfu. ^δ^ TTD = time to death. “S” denotes survivor. * *p*-value lower than significance level α = 0.05.

**Table 4:** Repeated measures ANOVA statistics for RR-interval.

Virus	Macaque	Sex	Dose *	TTD ^δ^	F-Statistic	*p*-Value
EEEV	M161-16	M	8.2	6	6.626	0.014 *
	M163-16	M	7.5	6	13.92	0.0004 *
	M160-16	M	7.0	S	3.420	0.073
	M162-16	M	5.7	S	2.227	0.376
VEEV	M164-16	F	7.1	S	49.07	<0.00001 *
	M165-16	F	6.9	S	175.9	<0.00001 *
	M170-16	M	6.3	S	15.65	<0.00001 *
	M171-16	M	6.0	S	183.0	<0.00001 *

Statistics and *p*-values for RR-interval for EEEV and VEEV-infected macaque cohorts. ^£^ Doses in log_10_ pfu. ^δ^ TTD = time to death. “S” denotes survivor. * *p*-value lower than significance level α = 0.05.

**Table 5:** Fundamental frequencies of EEEV and VEEV courses of infection.

Virus	Macaque	Sex	Pre-Infection Frequency	Post-Infection Frequency	Febrile Period Frequency	Recovery Period Frequency
EEEV	*161-16*	*M*	*0.395*	*0.879*	*0.571*	
	*163-16*	*M*	*0.242*	*0.769*	*0.549*	
	160-16	M	0.351	0.242		
	162-16	M	0.307	0.351		
VEEV	164-16	F	5.974 (0.373)	11.070 (0.275)	4.393 (0.275)	3.514 (0.220)
	165-16	F	5.974 (0.373)	11.070 (0.286)	4.568 (0.286)	3.514 (0.220)
	170-16	M	11.245 (0.351)	13.881 (0.434)	4.217 (0.132)	3.163 (0.099)
	171-16	M	11.245 (0.351)	13.705 (0.428)	5.271 (0.165)	3.163 (0.099)

This table documents fundamental frequencies observed in the ECG metric of RR-interval. Disease periods are separated into pre-infection, post-infection (outside of any febrile period), and febrile periods for EEEV VEEV. Recovery periods were also included when applicable, specifically in the case of the VEEV cohort. Fundamental frequencies are displayed in cycles per day (cpd). Values for VEEV are larger due to the extended time periods used for analysis; the values in parentheses are normalized to the first harmonic value for the purposes of comparison to EEEV frequency values. Italicized values represent animals with fatal courses of disease.

## Supplementary Materials

The following are available online at https://www.mdpi.com/2076-0817/8/4/240/s1, Supplemental Figure S1: Representative Poincaré Plots for Nonsevere EEEV Infection Cohort; Supplemental Figure S2: Representative Poincaré Plots for Severe EEEV Infection Cohort; Supplemental Figure S3: Representative Poincaré Plots for VEEV Infection Cohort; Supplemental Figure S4: Sex Differences Apparent in HRV in VEEV-infected Cohort; Supplemental Figure S5: Example ECG Trace; Supplemental Table S1. Supplementary Macaque Clinical Data; Supplemental Table S2: Electrocardiographic Metrics.
