# Supplementary material for: Electrocardiography Abnormalities in Macaques after Infection with Encephalitic Alphaviruses
Source: Pathogens. 2019 Nov 16;8(4):240. doi: 10.3390/pathogens8040240 (PMC6969904; doi:10.3390/pathogens8040240)
Supplement: Supplementary file 1 [file pathogens-08-00240-s001.zip › Supplemental Tables.docx]

**Supplemental Table A1.** Supplementary Macaque Clinical Data. Age in years. Wt1 (kg) refers to pre-infection weight, Wt2 (kg) refers to weight at exposure, and Wt3 (kg) refers to weight at necropsy (NW = not weighed). *Doses in log_10_ pfu. Plaque reduction neutralizing test (PRNT_80_) results at right.

| **Virus** | **Macaque** | **Sex** | **Age** | **Wt1.** | **Wt2.** | **Wt3.** | **Dose*** | PRNT_80_ |
| --- | --- | --- | --- | --- | --- | --- | --- | --- |
| **EEEV** | M161-16 | M | 7 | 4.5 | 5.6 | 5.3 | 8.2 | <16 |
|  | M163-16 | M | 7 | 5.5 | 6.6 | NW | 7.5 | <16 |
|  | **Mean** |  | **7** | **5.0** | **6.1** |  | **8.0** | **<16** |
|  | M160-16 | M | 7 | 5.1 | 6.6 | 7.0 | 7.0 | 1280 |
|  | M162-16 | M | 7 | 6.2 | 8.0 | 8.4 | 5.7 | >10240 |
|  | **Mean** |  | **7** | **5.7** | **7.3** | **7.7** | **6.8** | **>5760** |
| **VEEV** | M164-16 | F | 5 | 3.4 | 4.0 | 4.2 | 7.1 | 10240 |
|  | M165-16 | F | 5 | 3.1 | 3.5 | 3.6 | 6.9 | 5120 |
|  | M170-16 | M | 6 | 4.7 | 5.2 | 5.5 | 6.3 | 20480 |
|  | M171-16 | M | 7 | 5.0 | 5.0 | 5.2 | 6.0 | 20480 |
|  | **Mean** | **-** | **5.8** | **4.1** | **4.4** | **4.6** | **6.8** | **14080** |

**Supplemental Table A2.** Electrocardiographic Metrics. Abridged list of metrics under study.

| ***Electrocardiography Metric*** | ***Abbreviation*** | ***Significance*** | ***Units*** |
| --- | --- | --- | --- |
| Arrhythmic Beat Detection | BAD | The number of arrhythmic beats detected during a logging period. | *Count* |
| Heart Rate | HR | Number of heartbeats per minute. | *bpm* |
| Heart Rate Variability | HRV | RR-Interval matched with RR-Interval_n+1_, plotted as an ordered pair. | *ms, Unitless* |
| Maximum Voltage Derivative of R-Wave | MxdV | Maximum rate of change of ECG trace at R-Wave. | *mV/ms* |
| Noise in ECG Trace | Noise | Approximation of noise level in ECG cycle; root-mean-square value of derivative in a single ECG cycle. | *Unitless* |
| P-Wave Count | PCt | Number of P-waves counted in one sample. | *Count* |
| P-Wave Height | P-H | Height of P-Wave from isoelectric level. | *mV* |
| PR-Interval | PR-I | Interval of time between beginning of P-Wave to the beginning of the R-Wave. Can detect atrial conduction block. | *ms* |
| P-Wave Width | P-Width | Interval of time from the P-Wave to the end of the P-Wave. | *ms* |
| QaT | QATN | Interval of time between the Q-Wave to the peak of the T-Wave. | *ms* |
| QR-Interval | QR-I | Interval of time between the Q-Wave to the peak of the R-Wave. Indicator of ventricular depolarization and/or contraction. | *ms* |
| QRS complex | QRS | Interval of time between the Q-Wave to the beginning of the S-Wave. Indicator of ventricular depolarization and/or contraction. | *ms* |
| QRS Amplitude | QRSA | Amplitude of QRS complex from the isoelectric level. Indicator of ventricular contraction. | *mV* |
| QT-Interval | QT-I | Interval of time between the Q-Wave to the end of the T-Wave. Indicator of electrolyte status as well as speed of total cardiac repolarization. | *ms* |
| R-Wave Height | R-H | Height of the R-Wave from the isoelectric level. | *mV* |
| RR-Interval | RR-I | Interval from one R-Wave peak to the next R-Wave peak. | *ms* |
| ST-Elevation | ST-E | Height of the T-Wave at algorithmically-determined point between S-Wave and T-Wave, to detect myocardial infarction. | *mV* |
| ST-Interval | ST-I | Interval of time between the S-Wave and the end of the T-Wave. | *ms* |
| T-Wave Height | T-H | Highest point between the end of the S-Wave and end of the T-Wave. | *mV* |
| Peak of T-Wave | T-P | Peak of T-Wave relative to isoelectric level, between end of S-Wave and end of the T-Wave. | *mV* |
| T-Wave Peak-to-End | T-Pe | Time between the peak of the T-Wave to the end of the T-Wave; indicator of ventricular reperfusion. | *ms* |
