# Supplementary figures and images for: Electrocardiography Abnormalities in Macaques after Infection with Encephalitic Alphaviruses
Source: Pathogens. 2019 Nov 16;8(4):240. doi: 10.3390/pathogens8040240 (PMC6969904; doi:10.3390/pathogens8040240)

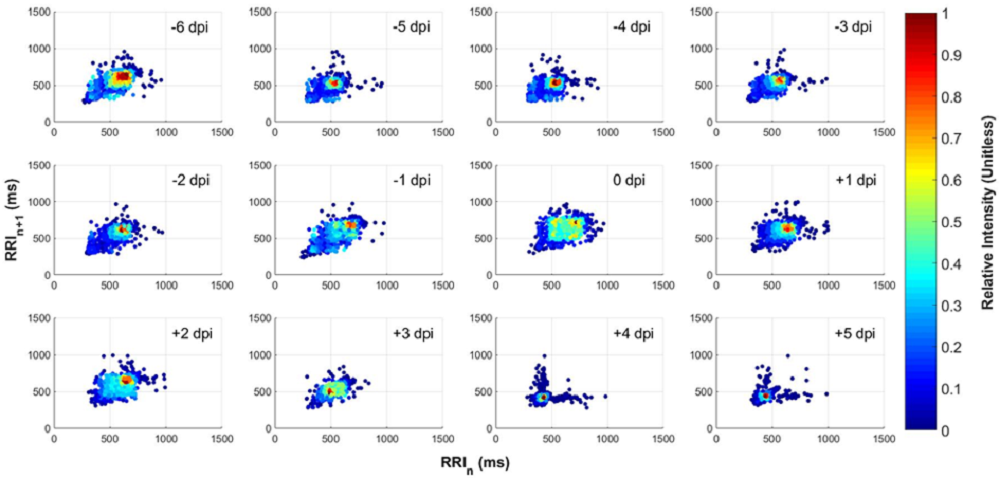

Supplement: Supplementary file 1 [file pathogens-08-00240-s001.zip › Supplemental_Revised/Supplemental Figure A2.png]

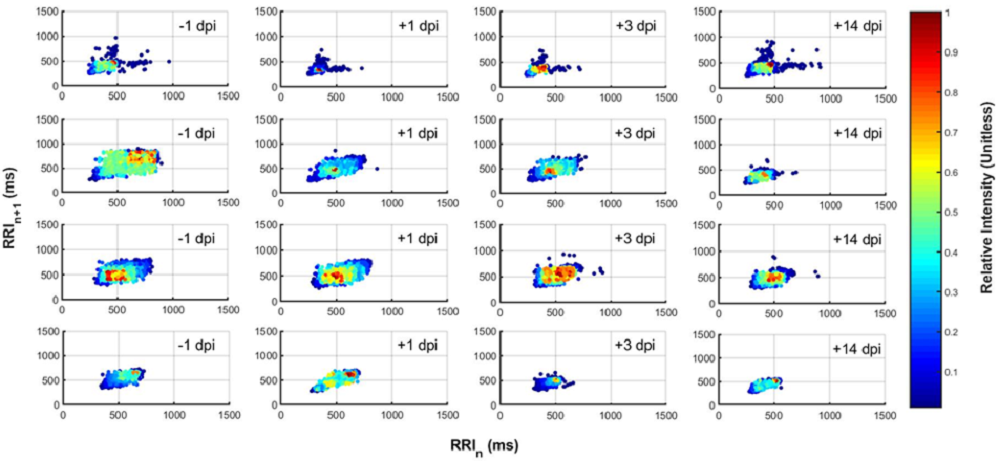

Supplement: Supplementary file 1 [file pathogens-08-00240-s001.zip › Supplemental_Revised/Supplemental Figure A3.png]

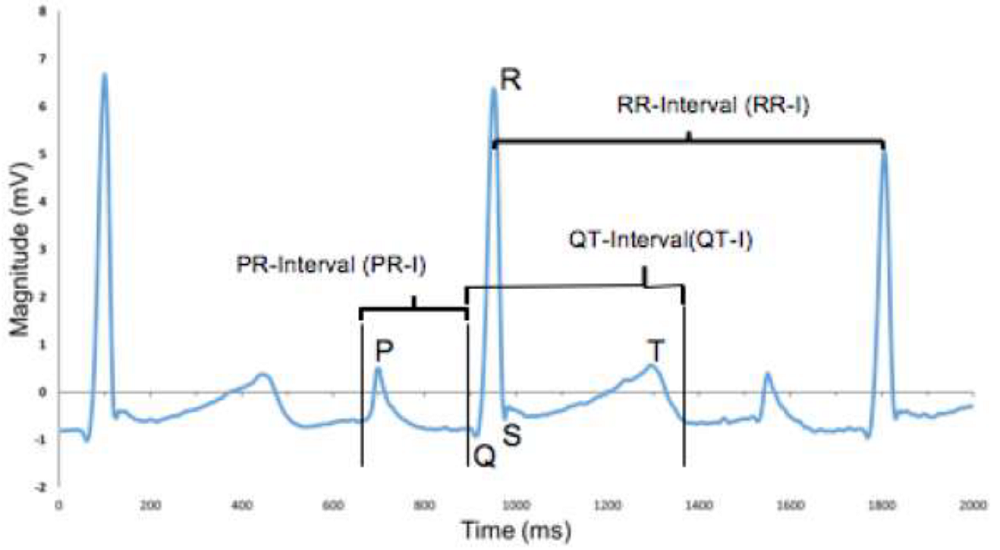

Supplement: Supplementary file 1 [file pathogens-08-00240-s001.zip › Supplemental_Revised/Supplemental Figure A5.png]

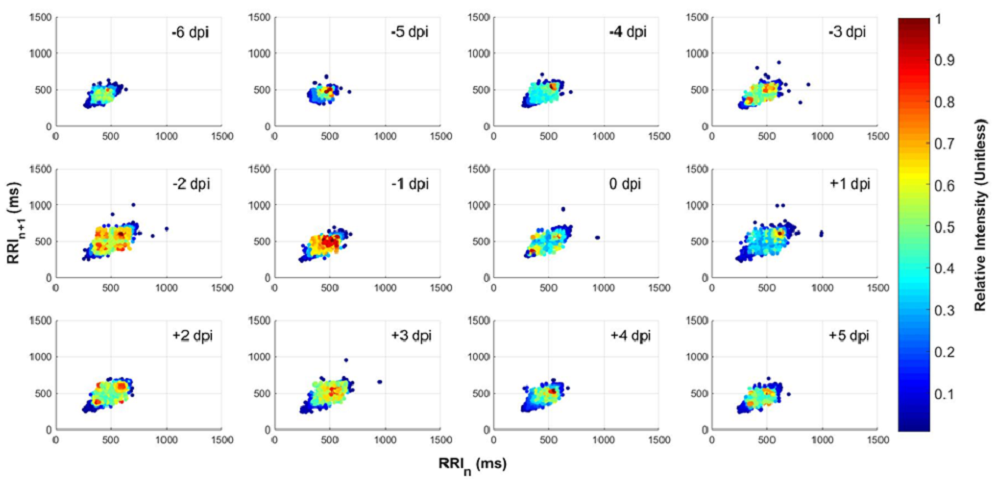

Supplement: Supplementary file 1 [file pathogens-08-00240-s001.zip › Supplemental_Revised/Supplemental Figure A1.png]

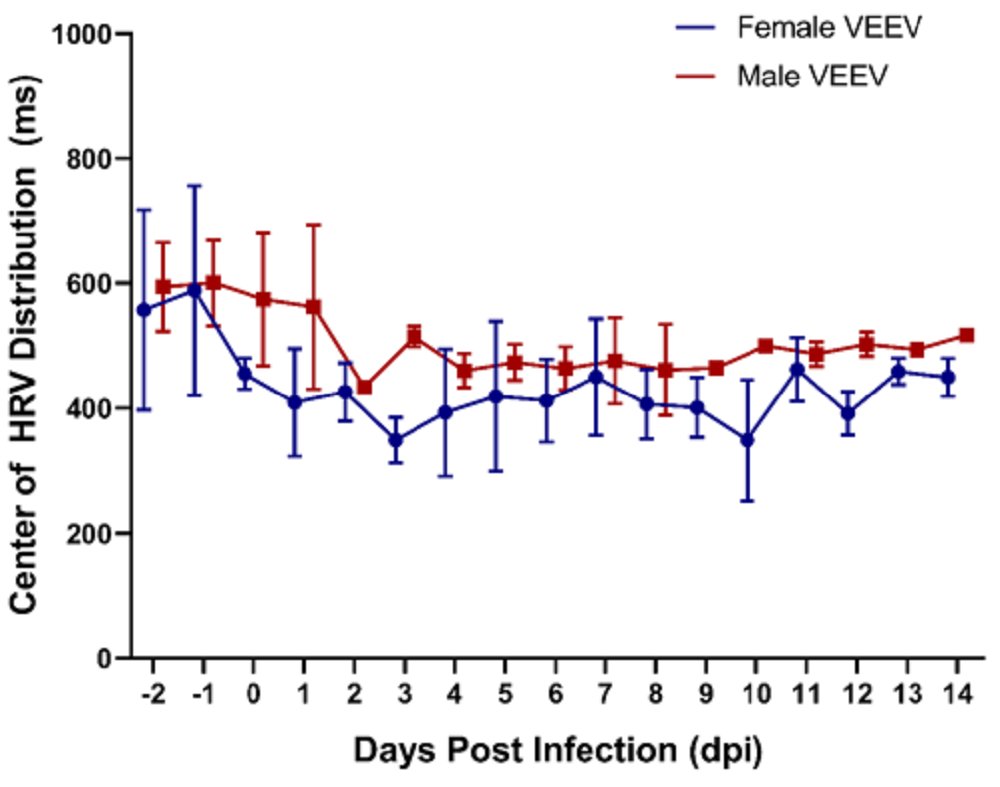

Supplement: Supplementary file 1 [file pathogens-08-00240-s001.zip › Supplemental_Revised/Supplemental Figure A4.png]
